# Diverse Microbial Hot Spring Mat Communities at Black Canyon of the Colorado River

**DOI:** 10.1007/s00248-023-02186-x

**Published:** 2023-02-09

**Authors:** Ivan J. Moreno, Bianca Brahamsha, Mohamed S. Donia, Brian Palenik

**Affiliations:** 1 Scripps Institution of Oceanography, University of California, San Diego, La Jolla, CA USA; 2 Department of Molecular Biology, Princeton University, Princeton, NJ USA

**Keywords:** Hot springs, Cyanobacteria, Leptolyngbya, Cyanobacterial interactions

## Abstract

The thermophilic microbial mat communities at hot springs in the Black Canyon of the Colorado River, thought to harbor the protistan human pathogen *Naegleria fowleri*, were surveyed using both culture-independent and -dependent methods to further understand the ecology of these hot spring microbiomes. Originating from Lake Mead source water, seven spring sites were sampled, varying in temperature from 25 to 55 °C. Amplicon-based high-throughput sequencing of twelve samples using 16S rRNA primers (hypervariable V4 region) revealed that most mats are dominated by cyanobacterial taxa, some but not all similar to those dominating the mats at other studied hot spring systems. 18S rRNA amplicon sequencing (V9 region) demonstrated a diverse community of protists and other eukaryotes including a highly abundant amoebal sequence related to *Echinamoeba thermarum*. Additional taxonomic and diversity metric analyses using near full-length 16S and 18S rRNA gene sequencing allowed a higher sequence-based resolution of the community. The mat sequence data suggest a major diversification of the cyanobacterial orders *Leptolyngbyales*, as well as microdiversity among several cyanobacterial taxa. Cyanobacterial isolates included some representatives of ecologically abundant taxa. A Spearman correlation analysis of short-read amplicon sequencing data supported the co-occurrences of populations of cyanobacteria, chloroflexi, and bacteroidetes providing evidence of common microbial co-occurrences across the Black Canyon hot springs.

**Supplementary Information:**

The online version contains supplementary material available at 10.1007/s00248-023-02186-x.

## Introduction

Thermophilic microbial mats have historically been studied as models for life on early earth, for their microbial community interactions and for the discovery of novel organisms that are uniquely fit for their thermal environment [1]. The diversity of microbes found within these types of mats has typically been analyzed with respect to abiotic environmental factors such as pH[2], temperature[3, 4], and biogeography[5]. While temperature and pH have been important factors when comparing diversity between microbial communities, recent studies have shown that these alone do not act as the sole drivers for community richness for hot springs at near-circumneutral pH and temperatures that allow for chlorophyll-based phototrophy[2, 6].

The development of DNA sequencing technologies to assess diversity of microbial communities, both prokaryotic and eukaryotic, has allowed us to greatly improve the taxonomic levels at which we see this diversity in a variety of environments[7]. The most commonly used marker genes include 16S rRNA and 18S rRNA, for prokaryotic and eukaryotic organisms, respectively[8–10]. Despite these advances, the eukaryotic microbial communities in extreme environments, such as those found in hot springs, remain vastly understudied even in recently published work [11–13]. Clustering ribosomal rRNA sequences at a 97% sequence identity or higher is a commonly used approach but makes it difficult to determine intraspecies nucleotide differences or the presence of distinct taxa within established bacterial groups[14]. By using full-length marker gene sequencing and computational methods that allow for denoising rather than clustering, or a combination of both [15], has recently made it possible to distinguish between closely related organisms at the single nucleotide difference level. In contrast, classical culture-dependent methods give researchers the ability to characterize microbes in more detail and test for fitness and other physiological properties, but it is well known that this approach has biases[16].

The hot springs found in Black Canyon of the Colorado River along the border of NV and AZ, USA (located at 35.806383, − 114.707921) are part of the Lake Mead National Recreation Area and range from 25 to 63°C, all of which are near neutral pH[17]. The heat source of these hot springs is not fully understood, but Lake Mead, which is north of Black Canyon and the Hoover Dam, is thought to be the source of the ground water that seeps through the canyon walls and helps creates these thermal springs[17]. These springs are frequently visited by tourists as they feed into potential bathing pools [18]. However, the National Park Service has deemed the Black Canyon hot spring systems as unsafe because their temperatures are similar to those where the pathogenic amoeba, *Naegleria fowleri*, can be found [19]. Taken together, these factors make the Black Canyon hot springs and microbial mats interesting ecosystems to explore microbial diversity and potentially bacterial-protist interactions.

Based on previously performed prokaryotic studies in fresh water hot springs, we expected that there would be broadly abundant cyanobacteria belonging to a single genus found across sites and especially at those sites within close distance of each other. This would be the paradigm based on research on hot springs in Yellowstone National Park. At Yellowstone multiple populations of *Synechococcus*, for example, are found at temperatures of up to 70 °C in different parts of the mats [5, 20, 21], although other cyanobacteria have been shown to be important in other hot spring systems [22]. We used a combination of methods including high-throughput amplicon sequencing and barcode-mediated full-length marker gene sequencing to describe the prokaryotic and protistan communities found at the freshwater springs in Black Canyon, as well as culture-dependent methods to characterize cyanobacterial ecosystem members.

## Materials and Methods

### Sample Collection

The thermal springs cyanobacterial communities found at Black Canyon exist mostly as thin films in streams of water seeping from the canyon walls, although some have a thicker mat-like structure. Collection of microbial mat samples was conducted in September of 2016. Microbial mats were sampled at a total of seven sites: Weeping Cave (3A), Main Pool (6A), Site 8 (8A), Boyscout Canyon (9A, 9B, 9C), Moonscape (10A), Arizona Hot Springs—Middle Pool (13A, 13B, 13C), and Arizona Hot Springs—Upper Pool (13D, 13E). Mat sample sites were selected based on the number of springs found at each collection site, with the hot spring site named Arizona Hot Spring containing the most extensive thermal pool and microbial mat system. Sample 10 was collected from a much lower temperature microbial mat environment as it served as a naturally occurring non-thermal control. Mat samples were collected and immediately preserved in Thermofisher RNAlater stabilization solution at a volume ratio of 1:5 to preserve the nucleic acids of the cells in sterile 5-ml cryovials. Additional mat samples were collected in September 2015 and 2016 for cyanobacterial isolation. The surface temperature of the water found at each sampling site was recorded using a Etekcity LaserGrip 774 infrared thermometer (www.etekcity.com).

### DNA Extraction and Sequencing

Mat samples previously stored in RNAlater at – 20 °C were transferred into 2.0-ml microcentrifuge tubes. All samples with the exception of 13A and 13B were centrifuged at 13,500 rpm and the RNAlater was decanted. This was repeated until the entire mat sample was pelleted within the tube and all RNAlater had been removed. DNA extraction was performed on samples by first incubating at 37 °C for 30 min after adding 560 μl of TE buffer and 80 μl of 100 mg/ml of lysozyme. This was followed by adding 80 μl of 10% SDS and 80 μl of proteinase K and then incubating once more at 55 °C for 2.5 h. A last incubation used 37 °C for 30 min after adding 16 μl of 10 mg/ml RNAseA. Extractions were completed using a phenol-chloroform extraction as follows: nucleic acids were extracted twice with an equal volume of 25:24:1 phenol:chloroform:isoamyl alcohol and once with 24:1 chloroform:isoamyl alcohol. The phenol-chloroform saturated the entirety of each of the mat samples. The nucleic acids in the aqueous phase were removed and then further purified using a DNeasy Blood and Tissue kit from Qiagen. The rest of the purification protocol was followed according to the manufacturer’s specifications and then eluted using 200 μl of the buffer provided in the kit. Samples 13A and 13B were processed for DNA extraction using the Qiagen PowerSoil kit and eluted using 100 μl of nucleic acid–free water. All DNA eluted was then quantified using a Nanodrop ND-1000 Spectrophotometer. Following quantification, library preparation and paired-end amplicon sequencing were conducted by Research and Testing Laboratories (Lubbock, TX). Modified bacterial and archaeal-specific primers and the eukaryotic primers from the Earth Microbiome Project were used for amplification[23]. These primers, 515yF (5′-GTGYCAGCMGCCGCGGTAA-3′) and 806bR (5′- GGACTACNVGGGTWTCTAAT-3′) [24], Euk1391F (5′-GTACACACCGCCCGTC-3′)[25], and EukBR (5′-TGATCCTTCTGCAGGTTCACCTAC-3′)[26], targeted the V4 and V9 hypervariable regions of the 16S and 18S rRNA genes, respectively. Both 16S and 18S rRNA amplicon sequencing was performed on an Illumina MiSeq platform. Full-length ribosomal marker gene sequencing and the accompanying library preparation methods were performed by Loop Genomics using the protocol for the kit LoopSeq^TM^ 16S and 18S long-read (loopgenomics.com)[27]. Amplification of 16S rRNA and 18S rRNA genes was performed using several forward primers (5′-AGAGTTTGATCMTGGCTCAG-3′, 5′-GATTAAGCCATGCAAGTS-3′, 5′-GGTTGATYCTGCCRGTRG-3′, 5′-TCYGGTTGATCCTGCC-3′) as well as two reverse primers (5′-GMWACCTTGTTACGACTT-3′, 5′-TACCTTGTTACGACTT-3′).

### Data Processing and Analysis

Raw sequences, both amplicon and full-length, were analyzed using the QIIME2 pipeline[28]. Merging, denoising, and filtering of amplicon paired-end sequences were performed using the DADA2[15] plug-in available on QIIME2 2019.4 with paired options to create amplicon sequence variants (ASVs). The full-length ribosomal sequences were denoised, quality checked, and filtered, and LASVs (long amplicon sequence variants) were created using DADA2 version 1.16 as a standalone tool in R 4.0. Bacterial sequences were retained using the primer detection parameter by searching for the 27F primer (5′-AGAGTTTGATCMTGGCTCAG-3′) used during sequencing[29]. The parameter DETECT_SINGLETONS=TRUE was used to retain single-sequence-count ASVs of high quality generated using Loop Genomics full-length 16S rRNA sequencing kit. Full-length ribosomal sequence data were imported into QIIME2 to perform diversity and taxonomic analysis. Taxonomic classifications of the ASVs for the 16S rRNA amplicon sequences, as well as the LASVs, were assigned using the 99% identity representation sequences from the SILVA 132 database [30]. The 18S amplicon sequences were assigned using the Protist Ribosomal Reference Database version 4.11.1 [31]. Full taxonomic classifications were used, with metazoans included, to identify the most abundant metazoans. Data used for constructing taxonomic bar plots was created after filtering out metazoans to identify the most abundant protists with sequences classified as unknown included. A principal coordinate analysis (PCoA) using unweighted unifrac[32] was performed for the 16S amplicon dataset using the diversity plugin and visualized using Emperor [33].

Alpha diversity of communities was assessed using all DNA sequences retained after quality control and alignments for both the 16S and 18S rRNA amplicon-based sequencing. ASVs and LASVs observed were used as a metric for comparison between different samples and sequencing methods, in addition to Shannon diversity and Faith’s phylogenetic distance indices calculated for short-read ASV datasets. Rarefaction was performed to create evenly sequenced depths across all samples by randomized subsampling at a depth of 9000 sequences which was sufficient to include all prokaryotic sample datasets. To compare all six samples used for full-length based prokaryotic community analysis to all 12 samples for the 16S rRNA amplicon-based sequencing, the cut off for rarefaction in the full-length sequencing reads was 6000 sequences. Data used for beta-diversity analysis between the short-read amplicon sequences was obtained after rarefaction at 9000 sequences. Reads based on 18S rRNA amplicon-based sequencing were rarified at 3000 sequences to include all 10 samples used for protist alpha diversity community analysis. Taxonomic assignments of the most abundant sequences in each sample after filtering for retainment of solely protists were performed with the NCBI BLAST tool on September of 2022. It should be noted that we followed the suggested QIIME2 pipeline and the integrated DADA2 program workflow where sequences were clustered at 100% identity and each cluster annotated as an ASV. This can result in a small number of ASVs with 100% identity over their aligned length but of varying lengths being assigned different ASV numbers. Sequences with varying lengths were the result of the denoising process of DADA2 that retains base pairs based on quality scores, so any nucleotides at the end of sequences that did not meet quality control thresholds were trimmed at that base pair. These sequences of varying length were also retained to increase phylogenetic resolution when assigning taxonomy.

Lastly, a Pearson correlation analysis was performed across all amplicon 16S rRNA libraries using the Cytoscape plug-in program CoNet version 1.1.1 [34]. Settings for this analysis were set to include only co-occurrences of ASVs with a Pearson correlation score of ≥ 0.85 and with a *P*-value threshold of ≤ 0.05. Parameters adjusted within this program analysis included standardization of reads per sample and a subsampling method of 1000 iterations of bootstrap analysis. All ASVs with an abundance of 1% or more per sample along with their associated correlations were then extracted from the data table. The results were then visualized using Cytoscape 3.7.1 and included ASVs displayed as nodes with taxonomy at the phylum level labeled by color. Weight of the correlation between two ASVs was displayed using increasing line width. All nodes with 10 or more direct connections to other nodes were centered within their respective networks and are discussed within this study as “central nodes.”

### Cyanobacterial Isolation and Identification

Microbial mat samples and accompanying spring water were brought back to the lab in sterile polycarbonate bottles. Once at the lab, pieces of the mat were streaked for colony formation on BG11 agar plates[35] and incubated at 30 °C with continuous broad-spectrum fluorescent lighting. Single filaments or colonies were picked from the plates and then cultured in BG11 liquid growth medium. DNA was purified from isolated colonies using a boil prep DNA extraction followed by purification using the DNeasy Blood and Tissue kit from Qiagen. Cyanobacterial specific primers CYA106F (5′-CGGACGGGTGAGTAACGCGTGA-3′) and CYA781R (5′-GACTACWGGGGTATCTAATCCCWTT-3′) [36] were used to amplify regions of the 16S rRNA gene using the following amplification conditions: 94 °C for 5 min to denature DNA, 94 °C for 1 min 29 times, each time followed by 50 °C for 1 min, 72 °C for 1 min, and then 72 °C for 7 min for the final extension after the last cycle. Amplified DNA was then stabilized at 4 °C until the PCR was manually terminated. The amplified DNA was sent for Sanger sequencing to Eton Biosciences (San Diego, CA) to be sequenced using the same primers used for amplification. Sequencing results from both forward and reverse strands were checked for nucleotide quality using FinchTV 1.4 (geospiza.com/finchtv), assembled, and consensus sequences for all isolates were obtained. Sequences were aligned and trimmed on CLC Genomics Workbench 12. The resulting sequences were trimmed to 543 aligned base pairs and were used to create a phylogenetic tree produced using the neighbor joining tree construction method and Jukes-Cantor model for nucleotide distance measures with 1000 replicates on CLC Genomics Workbench 12. The most abundant cyanobacterial ASVs and LASVs (determined below) were also included for phylogenetic analysis. To detect for the potential presence of each isolate within our ASV and LASV datasets, relative abundance and BLAST hits were inferred using the create BLAST database tool [37] and BLAST to custom database on CLC Genomics Workbench 12 (https://www.qiagenbioinformatics.com). The isolate was counted as “present” when there was a 100% identity match in the database. The number of matches between isolate and LASV sequences across samples was noted and added to the phylogenetic tree of all

cyanobacteria detected using long-read sequencing. The closest identity hit of each isolate was also identified using the NCBI BLAST database results from September 2022 [37].

## Results

### Sample Collection

**Fig. 1:**
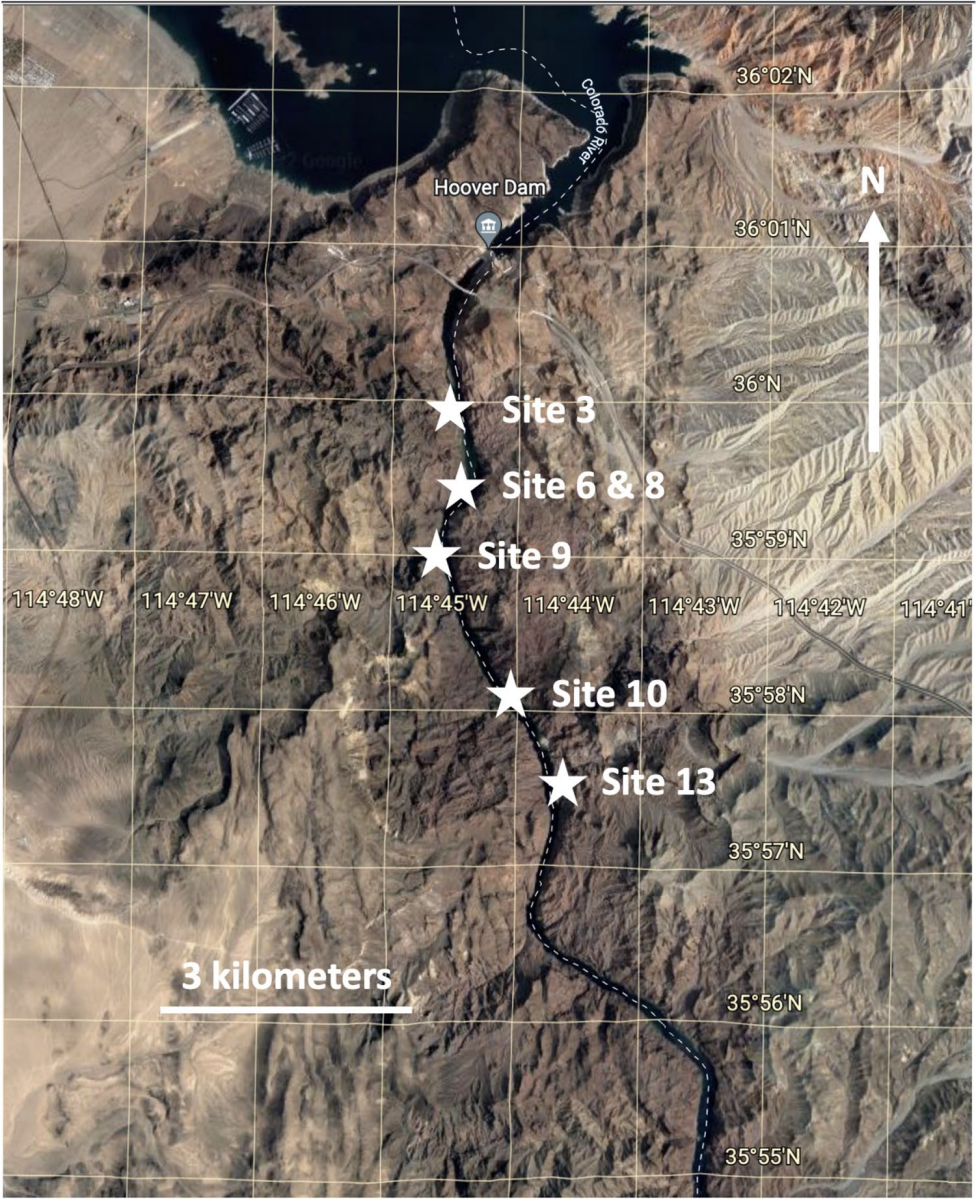
Map of Black Canyon of the Colorado River with the sites of mat samples. The numbers seen in the sample names correspond to the site numbers seen on the map. North of the Colorado River is Lake Mead, the expected source of the water that forms the hot springs at the various sites listed. Map was created using Google Earth

**Fig. 2:**
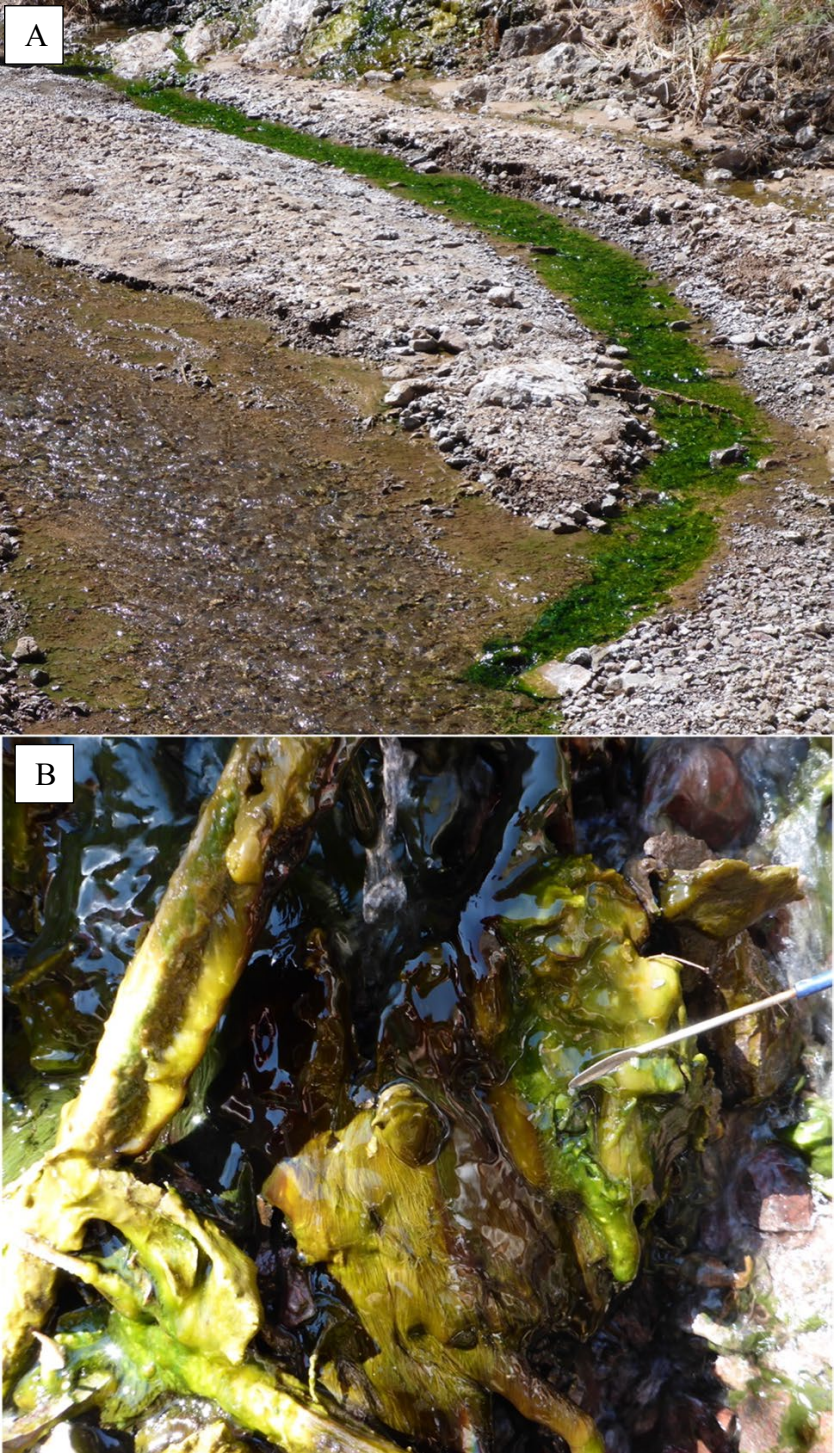
Hot spring microbial mat morphology seen at Boyscout Canyon in the Black Canyon of the Colorado River. **A** The source of the mat is a seep in the top left with a stream of hot water entering a cold-water stream at the bottom right. A thin green mat is found in the hot water stream (samples 9A and 9C). **B** A complex mat is found at the seep itself (sample 9B)

The temperatures at which each sample was collected and GPS coordinates of each site can be seen in Table 1. The geographical location for each spring at which samples were collected is shown on the map in Fig. 1. The morphology of the mats from which the samples were obtained varied across sites from thin “films” to laminated mats. Mats were typically green in color as seen in the mat found at site 9, Boyscout Canyon (Fig. 2A, B), but sites also included red or purple mats due to different cyanobacterial pigments. Overall, the cyanobacterial mats showed a high degree of morphological (color, texture, etc.) diversity.

**Table 1:** Mat collection sites. Names of each sample collected, along with their respective site of collection, the temperature at which the mat was found, and the type of sequencing performed for each sample

**Site**	**GPS coordinates**	**Sample**	**Temperature (°C)**	**Sequencing performed**
Site 3 Weeping Cave	36.00159, − 114.74603	3A	37.5	16S/18S
Site 6 Main Pool	36.0008, − 114.742476	6A	46.4	16S/18S
Site 8 Fluorescent Green Pool	36.00000, − 114.743483	8A	47.8	16S/18S/long read
Site 9 Boyscout Canyon	35.984774, − 114.744984	9A	39.4	16S/18S/long read
Site 9 Boyscout Canyon	“ “	9B	55	16S/18S/long read
Site 9 Boyscout Canyon	“ “	9C	40.8	16S/18S/long read
Site 10 Moonscape	35.952444, − 114.739361	10A	25	16S/18S
Site 13 AZ Hot Springs—Middle	35.960434, − 114.725546	13A	43.3	16S
Site 13 AZ Hot Springs—Middle	“ “	13B	40.1	16S
Site 13 AZ Hot Springs—Middle	“ “	13C	39.4	16S/18S
Site 13 AZ Hot Springs—Upper	35.964860, − 114.714429	13D	50	16S/18S/long read
Site 13 AZ Hot Springs—Upper	“ “	13E	50	16S/18S/long read

### Molecular Diversity of Prokaryotic Communities

The samples and their amplicon sequence variants obtained using both short amplicon (ASVs) and full-length sequencing (referred to here as LASVs) were found to have varying alpha diversity values seen in Table 2 for ASVs and compared to LASVs as observed ASVs in Supplementary Fig. 1. At the sequence subsampling depths seen in Supplementary Fig. 1, the lowest number of 16S rRNA ASVs observed was in sample 6A (53 ASVs) from main pool and samples 9A (88 ASVs) and 9B (114 ASVs) from Boyscout Canyon. At the same rarefaction subsampling depth of 6000 sequences, all other 16S rRNA communities had greater than 160 observed ASVs. As expected, there was generally a higher number of LASVs observed using full-length marker gene sequencing (Supplementary data Tables 1 and 2).

**Table 2:** Sample descriptions and alpha diversity metric values. Prokaryotic and eukaryotic Shannon diversity metric values for all samples as well as observed ASV and Faith’s phylogenetic distance values for prokaryotic communities. Distance from Lake Mead, the suspected source of water for these springs. Values for prokaryotic community diversity indices calculated based on subsampling normalization of sequencing data at a depth of 9000 sequences and those for eukaryotic sequencing at 3000 sequences

**Site and sample**	**Distance from Lake Mead (km)**	**Prokaryotic Shannon diversity**	**Prokaryotic observed ASVs**	**Prokaryotic Faith’s phylogenetic distance**	**Microbial eukaryote Shannon diversity**
3A	1.53	4.84	605.9	57.04	3.21
6A	1.33	2.82	53	12.87	1.89
8A	1.61	4.15	171.7	25.82	2.99
9A	3.38	3.34	88.1	16.26	1.03
9B	3.38	3.73	113.5	19.22	2.5
9C	3.38	7.77	797.2	71.88	2.91
10A	8.08	5.8	242.5	23.36	1.91
13A	6.63	6.02	670.7	63.1	
13B	6.63	4.19	619.2	61.64	
13C	6.63	5.23	304.6	37.51	3.23
13D	7.85	7.37	606	55.9	1.8
13E	7.85	4.17	170.5	26.16	3.42

**Fig. 3:**
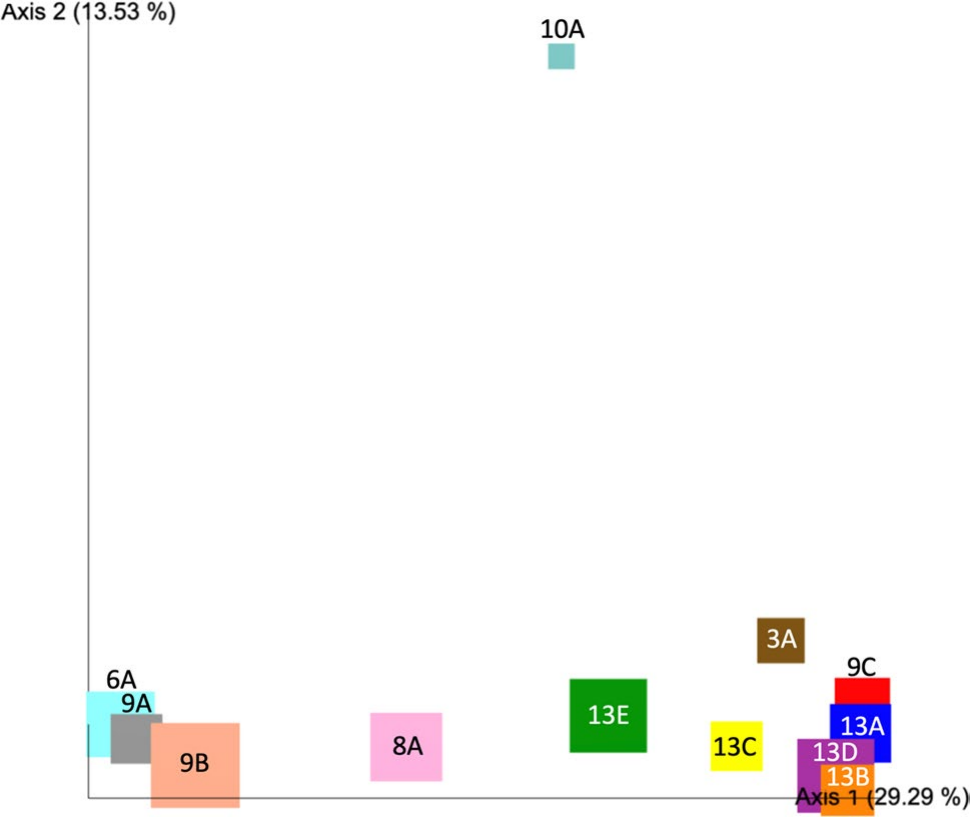
Unweighted-unifrac-based PCoA to identify community composition across temperatures of sites where mat samples were collected. The phylogenetic differences between all 16S rRNA amplicon-based samples, with sites represented by color. Temperature is denoted by size of square by making the smallest square the lowest temperature and vice versa. The protist *Echinamoeba thermarum* was found in samples 6A, 9A, and 9B, and the abundant eukaryote *Chaetonotus* was found in samples 3A, 9C, and 13D, clustered on the left and right, respectively

The community composition and beta-diversity of the V4 amplicon gene sequences were compared using an unweighted-unifrac distance metric-based principal coordinate analysis (PCoA) while subsampling at a depth of 9000 sequences (Fig. 3).

### Diversity of Cyanobacterial Communities

Each mat sample was typically dominated by one distinct cyanobacterium (Table 3), apart from the sample from site 10A which was collected from an exposed willow root as described above. In multiple sites, the most abundant cyanobacterium was not closely related to any cyanobacterial isolate sequences found in the Silva 132 database resulting in the annotation “uncultured sp.” shown in Fig. 5A. The most abundant cyanobacterial ASVs (V4 amplicon sequencing) were found to be in similar relative abundance to the most abundant LASVs in the same samples (Table 3). These were usually filamentous cyanobacteria from diverse genera such as *Leptolyngbya*, *Oscillatoria*, *Phormidium*, etc., with sometimes different top BLAST hits (Table 3).

**Table 3:** Most abundant cyanobacteria per sample. Comparisons between dominant cyanobacterial ASV sequences generated from short-read amplicon sequencing and their closest related LASV sequences generated using long-read

**Sample**	**Most abundant ASV**	**% Sequence abundance**	**Most abundant LASV**	**% Sequence abundance**	**Closest isolate BLAST hit of ASV vs LASV**
3A	ASV925	54.13	N/A	N/A	*Oscillatoria sp. DV-00-5*
6A	ASV1808	45.76	N/A	N/A	*Synechoccocus sp. OH4*
8A	ASV3151	47.38	LASV2	36.97	*Leptolyngbya sp. PKUAC-SCTB412* vs *Phormidium sp. NIES-2120*
9A	ASV4406	38.39	LASV1	28.49	*Phormidium sp. SC-S3a* vs *Leptolyngbya sp. PKUAC-SCTB412*
9B	ASV2506	27.46	LASV1	29.96	*Thermoleptolyngbya sichuanensis PKUAC-SCTA183* vs *Leptolyngbya sp. PKUAC-SCTB412*
9C	ASV3179	14.25	LASV8	7.74	*Aerosakkonema funiforme strain ACKU622* vs *Pseudanabaena sp. 63-1*
10A	ASV4402	11.65	N/A	N/A	*Romeria sp. BPE57*
13A	ASV1044	24.54	N/A	N/A	*Neolyngbya sp. OdA1*
13B	ASV2028	56.28	N/A	N/A	*Oscillatoria sp. DV-00-5*
13C	ASV483	27.49	N/A	N/A	*Neolyngbya sp. OdA1*
13D	ASV1591	4.01	LASV22	5.32	*Leptolyngbya sp. WR9* vs unclassified
13E	ASV437	29.28	LASV3	19.27	*Phormidium sp. NIES-2120 vs Thermoleptolyngbya sichuanensis PKUAC-SCTA183*

While short-read amplicon sequencing revealed multiple samples in which a single ASV was responsible for nearly the entire relative abundance of the cyanobacterial population, in some instances, different ASVs represented the same genus or species. In a few cases the differences between two ASVs were length differences likely due to differences in sequence quality. This was the case in samples 3A (ASV925) and 13B (ASV2028), where the most abundant cyanobacteria in both samples and their ASV sequences have 100% identity in the region of overlap but a difference of 8 nucleotides in length at the end of the ASV 2028 (Fig. 5, Supplementary data Table 1). Similarly, samples 13A (ASV1044) and 13C (ASV483) were from unknown cyanobacteria of the same order represented by single ASVs with 100% identity to each other over their aligned region but of different sequence lengths. The assignment of different ASVs to what may or may not be the same cyanobacterium is due to our use of the standard QIIME2 pipeline workflow (see methods). Samples, 9A and 10A, however, also had single ASVs, ASV4406 and ASV4402, respectively, which were taxonomically classified as belonging to the same taxon, but these two ASVs were distinct and had only 86% nucleotide identity across amplicons of 263 base pairs.

### Taxonomic Classification and Diversity of Non-Cyanobacterial Prokaryotes

**Fig. 4:**
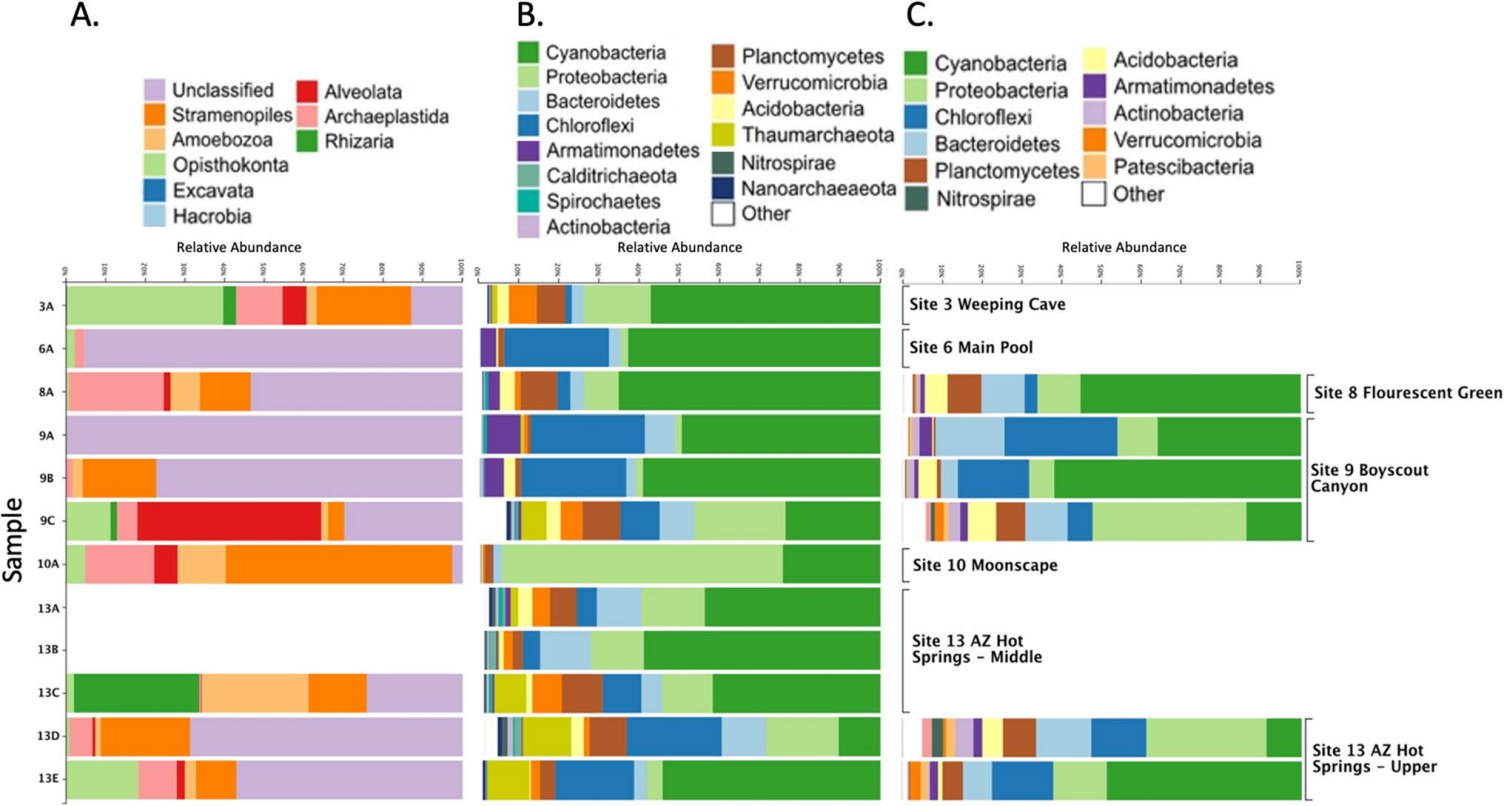
Microbial community composition. **A** Protist community composition shown as bar plots indicating the classification results using the PR2 database. **B** Prokaryotic community composition shown as bar plots indicating the classification using the 16S rRNA SILVA 132 database. **C** Taxonomic classification results of the long-read 16S and 18S rRNA sequencing. All ASVs were classified using the full SILVA 132 database which includes both 16S and 18S rRNA sequences. Each row of taxonomic barplots represents a single sample; thus those for which only a single type of sequencing was performed only have a single barplot in that row (i.e., samples 13A and 13B)

Using short amplicon sequencing, nine samples (3A, 6A, 8A, 9A, 9B, 13A, 13B, 13C, and 13E) had a 40% or greater 16S rRNA relative read abundance of cyanobacteria with the remainder of reads being from diverse bacterial taxa. Samples 13D and 9C had a lower abundance of cyanobacteria (10% and 24%, respectively) and the two had similar levels of other bacteria (Fig. 4B).

Sample 10A, from a submerged willow root downstream of a hot spring, although somewhat atypical, had a 70% relative read abundance of proteobacteria with the largest abundance within that population being ASV565 (Supplementary data Table 1) that was taxonomically classified as the proteobacteria genus *Hydrogenophaga*.

Chloroflexi were abundant in the mat samples, with the most abundant being from the Anaerolinaeae group A4b or RBG-13-54-9 (Table 4). Sample 10A was unusual with the most abundant Chloroflexi ASV and LASV being a *Herpetosiphon* sp.-related sequence.

**Table 4:** Table of most abundant ASVs and LASVs belonging to the group Chloroflexi, the second most abundant group across all samples. Percent of relative abundance of Chloroflexi sequences in each sample and closest NCBI BLAST hits and classifications based on the Silva 132 database included for each ASV and LASV. All classifications except for that in sample 10A fall within Anaerolineae

**Sample**	**Most abundant ASV**	**% Relative ASV abundance**	**Most abundant LASV**	**% Relative LASV abundance**	**Closest BLAST Hit**	**Silva 132 database classification (ASV vs LASV)**
3A	ASV3508	10.17%	N/A	N/A	Uncultured	SBR1031; A4b
6A	ASV984	5.76%	N/A	N/A	Uncultured	RBG-13-54-9
8A	ASV2699	5.32%	LASV4	0.32%	Uncultured	SBR1031; A4b vs RBG-13-54-9
9A	ASV984	15.59%	LASV4	10.17%	Uncultured	Both RBG-13-54-9
9B	ASV984	0.50%	LASV4	8.08%	Uncultured	Both RBG-13-54-9
9C	ASV2699	0.87%	LASV12	0.31%	Uncultured	SBR1031; A4b vs Anaerolineaes; Anaerolineaceae
10A	ASV1872	2.10%	N/A	N/A	Herpetosiphon sp. OSI-B2	Chloroflexia; Chloroflexales; Herpetosiphonaceae; Herpetosiphon; Herpetosiphon aurantiacus DSM 785
13A	ASV292	0.21%	N/A	N/A	Uncultured	SBR1031; A4b
13B	ASV3860	16.99%	N/A	N/A	Uncultured	SBR1031; A4b
13C	ASV2699	10.40%	N/A	N/A	Uncultured	SBR1031; A4b
13D	ASV3385	1.13%	LASV17	2.47%	Uncultured	SBR1031; A4b vs Anaerolineaes; Anaerolineaceae; UTCFX1
13E	ASV4094	0.08%	LASV12	4.56%	Uncultured	Both Anaerolineaes; Anaerolineaceae

Another notable group of bacteria found at high relative sequence abundance includes a Bacteroidetes ASV171, classified only to order level and found most abundant in sample 13B. It was also 7% of the total relative sequence abundance in the short-read libraries (Supplementary data Table 1). This same sequence was not similar to known bacterial isolates in a search on the NCBI database, with the closest match only having an 87% identity. Other Bacteroidetes were also found and are likely important as seen in the network analysis (see below).

Samples 13A and 13C had a taxonomically well-defined population belonging to the family *Pedosphaeraceae* of the Verrucomicrobia with relative abundances of 7% and 4%, respectively, in the short-read amplicon libraries, represented by identical or nearly identical ASVs 1837 and 208.

Community composition inferred using the full-length sequencing of 16S rRNA genes revealed similar taxonomic bacterial population proportions at phyla levels compared to using the previously mentioned hypervariable V4 region. Unsurprisingly, the taxonomic classifications for the sequences in the full length 16S RNA data were resolved to species or genus taxonomic levels more frequently than using short-read amplicon sequencing (Supplementary data Tables 1 and 2).

### Co-occurrence Network

A co-occurrence analysis was performed to determine the prokaryotic microbiome members frequently found present with the most abundant cyanobacteria; the latter are typically considered the major primary producers in these types of mat communities. Network analysis of the short-read sequence data processed and filtered as described above resulted in 10 major networks (Fig. 6) with each network broadly representing different samples with central nodes surrounded by minor nodes and organized by phyla. The largest network (seen in Fig. 6A) is representative of two samples collected from the same source of water and site, samples 13A and 13B. Three of the four cyanobacterial central nodes were found most abundant in sample 13A, and one found most abundant in sample 13B. All were classified as belonging to the order Nostocales with two having classifications at genus/species level—ASV 4064 as *Planktothrix* sp. and ASV 2028 as a *Roseofilum* AO1-A sp. The four proteobacterial central nodes were represented by ASV 787 and 217 (gammaproteobacteria) and ASVs 12 (alphaproteobacteria) and 2474 (deltaproteobacteria). Of the five bacteroidetes central nodes, one, ASV 2153, was classified as belonging to the family *Saprospiracaea* while the other ASVs belonged to the group *Microscillacaea*. The Chloroflexi central nodes were represented by ASVs 292 (Anaerolineales) and 3687 (Chloroflexales). The two Verrucomicrobia central nodes belonged to ASV 208 and ASV 1216. Verrucomicrobia central nodes were represented by ASVs best classified as members of the group *Pedosphaeraceae*. The two Planctomycete central nodes belonged to ASV 853, classified as part of the group *OM190* and ASV 32 belonging to the group *Gemmataceae*. The sole Archaeal main node belonged to ASV 3568, best classified as a Candidatus *Nitrosotenuis sp*.

Other networks found in the analysis are seen in Fig. 6 B–J. Several networks show a central cyanobacterial node, as well as a 1–3 Chloroflexi, and often a Bacteroidetes node as seen in networks B, C, and D. All cyanobacterial central nodes belong to an ASV classified as filamentous in morphology. Chloroflexi central nodes also fall into two distinct taxonomic categories: Chloroflexales and Anaerolineales. All Bacteroidetes central nodes belonged to the orders Chitinophagales and Cytophagales. The only other proteobacterial central node, seen in Fig. 6F, was best classified as belonging to the genus *Hydrogenophaga*.

Aside from the ubiquitous central nodes, minor nodes surrounding the central nodes in each network are made up of a large diversity of different Proteobacteria species, indicating their prevalence in each sample. As seen in smaller networks without central nodes, networks seen in Fig. 6G, H, and I have cyanobacterial nodes but without the significant number of connections to meet the threshold of a “central node.” These may not have been abundant enough in our samples to resolve their networks.

### Taxonomic Classification of Protists

All 18S rRNA-based amplicon datasets were filtered to only include protists and unclassified eukaryotes due to the use of the protest-specific database used to classify the sequences. Even after filtering to remove any ASVs classified as metazoans, more than half of the sequences remained unclassified in samples 9A (99%), 6A (95%), 9B (77%), 13E (69%), 13D (57%), and 8A (53%) (Fig. 4A and Supplementary data Table 3). The remaining protists were classified by the second highest taxonomic ranking, which include Stramenopiles, Amoebozoa, Alveolata, Archaeaplastida, Rhizaria, Opisthokonta, Excavata, and Hacrobia. Groups that made up a large proportion of sequences in specific samples included Stramenopiles*,* which made up 57% of the sequences in sample 10A; Opisthokonta, which made up 39% of the sequences in the sample 3A; Rhizaria*,* which made up 32% of the sequences in sample 13C; and Alveolata*,* which made up 46% of the sequences in sample 9C. While these were the most abundant groups of protists in each sample, it is important to note that half of the samples included a large (> 50%) number of sequences that were unclassified (Fig. 4A). Large proportions of unclassified eukaryotes in samples 6A, 9A, and 9B were the result of the high proportional abundance of a single ASV found in all 3 of these samples. Of the two eukaryotic sequences found in the full-length 18S rRNA sequencing data from sample 9B, one is potentially representative of the abundant and unclassified eukaryotic ASV of communities 6A, 9A, and 9B. This finding is based on an exact 56 base pair match between the V9 regions of the 18S rRNA amplicon of the abundant ASV and a match to the most abundant LASV generated from the 18S rRNA sequencing data. An NCBI BLAST search using the full length 18S rRNA sequence revealed a 99% match to the protist *Echinamoeba thermarum*, which was previously identified in two hot spring studies as a prominent and potentially ecologically important member of the microbial mat community[38, 39]. Thus, it is likely that the major unclassified eukaryotic ASV and associated LASV at our sites is this protist or a close relative. All other most abundant protistan members found in each sample were further classified by use of the NCBI BLAST tool and assignments are seen in Table 5. In addition we used BLAST to identify the most abundant metazoan sequences, and this identified *Chaetonotus*, a gastrotrich, as being abundant in our samples especially 3A, 9C, and 13D (Table 5).

**Table 5:** Most abundant protist and microbial eukaryote 18S rRNA ASVs. Nucleotide sequence taxonomic classifications of the most abundant sequences found across all samples in which 18S rRNA sequencing was performed. Individual ASVs were both searched for on the NCBI BLAST nucleotide database and classified using the PR2 database. Those denoted with an asterisk had BLAST results with a query cover below 50% and results from the LASV sequencing performed were inferred to assign taxonomy

**Sample**	**Protistan ASV**	**Most abundant protistan sequence BLAST result**	**Most abundant protistan sequence PR2 classification**	**Most abundant Metazoan ASV**	**Most abundant Metazoan BLAST result**	**Most abundant Metazoan PR2 classification**
3A	ASV46	*Pythium insidiosum sp.*	*Peronosporales*	ASV2	*Chaetonotus* aff oculifer	*Gastrotrich sp.*
6A	ASV1*	Echinamoeba thermarum*	Unclassified	ASV7	*Philodina sp.*	*Adineta vaga sp.*
8A	ASV32	*Nematostelium ovatum*	*Streptophyta sp.*	ASV10	*Eimeriidae* sp.	Unclassified
9A	ASV1*	Echinamoeba thermarum*	Unclassified	ASV9	Unclassified	Unclassified
9B	ASV1*	Echinamoeba thermarum*	Unclassified	ASV9	Unclassified	Unclassified
9C	ASV15	*Monocystis agilis sp.*	*Syncystis mirabilis sp.*	ASV3	*Chaetonotus* aff euhystrix	*Gastrotrich sp.*
10A	ASV6	Unclassified	*Embryophyceae sp.*	ASV29	*Chironomus* tepperi	*Simulium*
13C	ASV16	*Edaphoallogr-omia australica*	*Tubulinea sp.*	ASV13	*Edaphoallog-ramia* australica	*Allogromia laticollaris*
13D	ASV17	*Loxodes sp.*	Unclassified	ASV3	*Chaetonotus* aff euhystrix	*Gastrotrich sp.*
13E	ASV10	*Lobosea sp.*	Unclassified	ASV11	Eimeriidae sp.	*Enoplea sp.*

### Cyanobacterial Isolates

Eighteen cyanobacterial isolates were obtained in culture and a partial 16S rRNA sequence for each was obtained using cyanobacterial-specific primers. The closest taxonomic match for each was found using the NCBI BLAST 16S rRNA database (Table 6). Searching for each isolate’s sequence within a custom BLAST database created using the mat sample full-length 16S rRNA sequences, from six of the twelve samples, nine isolates were found to have notable abundances at 100% identity to sequences in specific mat samples (Fig. 7). Cyanobacterial isolates BC1502, BC1503, and BC1507 formed a cluster of strains (Supplementary Fig. 2) most closely related (98.87% match) to *Aerosakkonema funiforme* Lao26 (NR_114306 [40]) and were 100% identical to LASV193 in sample 9C (Supplementary data Table 2). Additionally, BC1604 was a closely related isolate differing by only 2 base pairs (Fig. 7 and Supplementary Fig. 2) which suggests some microdiversity at the Black Canyon within this species.

**Table 6:** Cyanobacterial isolates from Black Canyon. Closest nucleotide sequence identity matches of cyanobacterial isolates from Black Canyon mats to related isolates. Those in bold font indicate no closely related isolates (below 95% identity)

**Isolate ID**	**Site**	**Closest related isolate**	**Accession**	**Percent identity**	**Closest related isolate environment**
BC1501	Site 13	*Leptolyngbya sp. PUPCCC 112.22*	KM376991	100%	Hot spring
BC1502	Site 13	*Aerosakkonema funiforme strain Lao26*	NR_114306	98.87%	Estuary/reservoir
BC1503	Site 13	*Aerosakkonema funiforme strain Lao26*	NR_114306	98.87%	Estuary/reservoir
BC1504	Site 13	*Synechococcus moorigangaii CMS01*	**KX579040**	**94.56%**	**Mangrove estuary**
BC1505	Site 13	*Synechococcus moorigangaii CMS01*	**KX579040**	**94.56%**	**Mangrove estuary**
BC1506	Site 13	*Synechococcus moorigangaii CMS01*	**KX579040**	**94.56%**	**Mangrove estuary**
BC1507	Site 13	*Aerosakkonema funiforme strain Lao26*	NR_114306	98.87%	Estuary/reservoir
BC1601	Site 6	*Mastigocladus sp. TB-4*	MN966425	100%	Thermal springs
BC1602	Site 3	*Leptolyngbya sp. PUPCCC 112.22*	KM376991	100%	Hot spring
BC1603	Site 8	*Phormidium sp. PUPPCCC 112.22*	KM438197	99.77%	Hot spring
BC1604	Site 9	*Aerosakkonema funiforme strain Lao26*	NR_114306	98.87%	Estuary/reservoir
BC1605	Site 8	*Pseudanabaena sp. 72.1*	**FJ769798**	**92.78%**	**Lagoon mat**
BC1606	Site 8	*Oscillatoria duplisecta ETS-06*	AM398647	98.41%	Thermal springs
BC1607	Site 6	*Thermostichus lividus PCC 6715*	MF191713	99.55%	Thermal
BC1608	Site 13	*Phormidium sp. NIES-2120*	LC455613	98.64%	NIEHS collection
BC1609	Site 8	*Pseudanabaena sp. 72.1*	**FJ769798**	**92.78%**	**Lagoon mat**
BC1611	Site 10	*Romeria sp. BPE57*	MN909722	97.49%	Temperature tolerant
BC1618	Site 10	Calothrix sp. N42	KY548062.1	96.08%	Agricultural soils

**Fig. 5:**
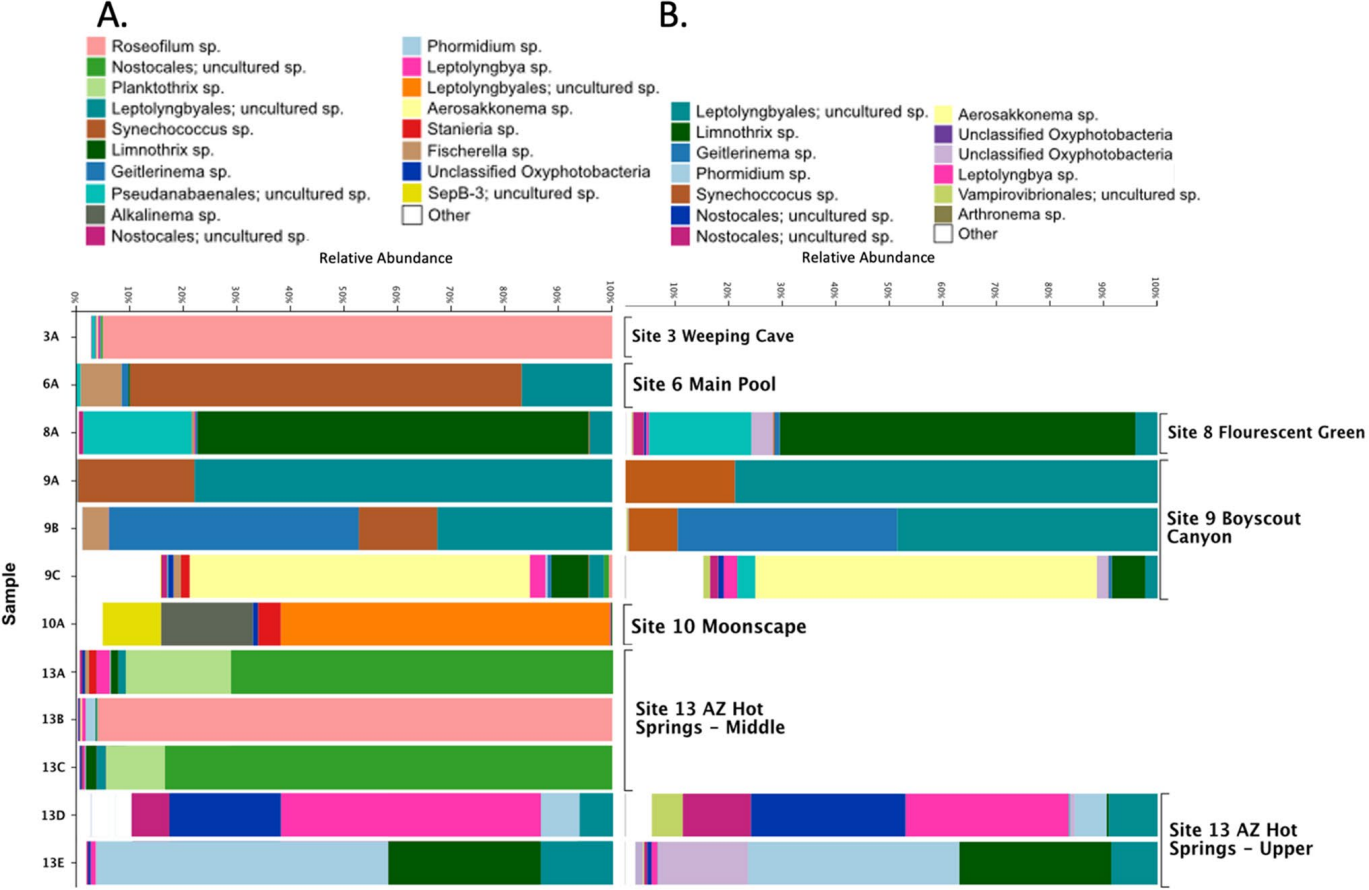
Comparison of taxonomic classification at the genus level from the use of two separate methods of high-throughput sequencing. **A** Cyanobacterial populations at the genus level. **B** Cyanobacterial populations in the same samples as (A), but with the use of full length 16S rRNA sequencing. Matching taxonomic classifications across sequencing methods were assigned the same color in both plots (A and B)

Isolate BC1608, which was most closely related to a *Phormidium* sp. (98.64% match), was found in high abundance in the cyanobacterial full-length 16S rRNA gene sequences in sample 13E (19%) (Supplementary Fig. 2). Having the predominant member of this community in culture should prove helpful in understanding its role in these microbial mats. Isolates BC1605 and BC1609 were 100% identical in the sequenced 16S rRNA region analyzed and were isolated from the same hot spring sample. They were both 92.78% identical to a *Pseudanabaena sp.* (accession FJ769798), which was found in a lagoon microbial mat. They were 100% identical to LASV13 and 645 full-length sequences were found in mat sample 8A (Supplementary data Table 2). Isolates BC1602 and BC1501 are 99% identical to each other and 100% and 99% identical to LASV1327, respectively, a low abundance cyanobacterium but detected in sample 9B. Isolate BC1602 was also 100% identical to the sequence of a *Leptolyngbya* sp. (KM376991), a known thermophilic cyanobacterium. Despite cyanobacteria from the group *Leptolyngbyaceae* being highly represented in sequence data from Black Canyon hot spring microbial mats, only a few were successfully isolated in this study (Table 6, Fig. 5).

## Discussion

In this study, we characterized the prokaryotic and eukaryotic communities of microbes found in 12 different mat samples in Black Canyon by using both high-throughput sequencing-based approaches, as well as culture-dependent methods (Fig. 4 and Table 6). The results were consistent when using either full-length or partial length amplicon marker gene sequencing (Fig. 4B and C; Tables 2 and 3). Of the 12 mat samples, most showed a different predominant cyanobacterium including those from the genera *Limnothrix*, *Geitlerinema*, *Leptolyngbya*, *Aerosakkonema*, *Phormidium*, an unknown Nostocales, *Synechococcus*, and *Roseofilum*. These results provide a different perspective on cyanobacterial populations in hot spring microbial mats relative to previously characterized microbial mats where *Synechococcus* and *Fischerella*/*Mastigocladus* species of cyanobacteria are typically found as the most abundant [6, 21, 41]. These results may be attributed to the lower temperatures found at Black Canyon hot springs that likely allow for a higher diversity of cyanobacterial mat populations and accompanying protists [42, 43]. Several cyanobacterial isolates were obtained (Table 6) that may be representative of some of the predominant members of the mats, and these should be valuable in future experimental work to identify possible adaptations contributing to their high relative abundance in each community.

### Microbial Mat Beta-Diversity

Environmental differences that could account for mat cyanobacterial diversity include abiotic factors such as water chemistry, water temperature, light intensity, stream flow rates, and gravel/rock type or biotic factors such as grazers and viruses. One possible reason for the diverse mat types is that even small temperature, water chemistry, and light differences create significant niche differences that partly account for the phylogenetic makeup of the microbial communities by favoring specific cyanobacterial genera, species, or ecotypes (Fig. 3). For example, a species of filamentous cyanobacteria, *Fischerella thermalis*, was noted to have evolved into multiple ecotypes, each of which is a variant found at specific temperatures ranging between 46 and 61 °C [41, 44].

The PCoA analysis in Fig. 3 shows a 29% variance across axis 1. One possible explanation for this may be the changes in water chemistry/distance from the water source (Lake Mead). As the composition of spring waters had been characterized previously by USGS, we did not obtain our own water samples for chemical composition, but it is known that the water geochemistry changes as it passes through the canyon. Levels of sulfate, chloride, calcium, sodium, and potassium are lower at Pupfish Hot Springs (0.2 miles from Hoover Dam) and are higher at site 9 in Boyscout Canyon, which is 2.2 miles from the dam, and site 13 in Arizona Hot Springs, which is 3.8 miles from the dam[17]. The effects of differences in elemental composition in the water of these springs may be notable in Fig. 3, where samples from site 9 and site 13 are on opposite sides of axis 1.

A “founder” hypothesis may also be important in explaining mat diversity. Mats may start with a founder population of a cyanobacterium that then has negative allelopathic strategies for reducing competition from other populations. Rain or flood events may wash away mats (observed in 2019, unpublished) and create new space that is then open for new colonization. This mechanism would create a patch work of mats somewhat like what was seen at Black Canyon. Evaluating this hypothesis would require cyanobacterial metagenomes and chemical allelopathy studies to detect distinct chemical toxin production as well as temporal monitoring or experimental manipulation of mat sites.

### Understanding the Role of Cyanobacteria in Mats

**Fig. 6:**
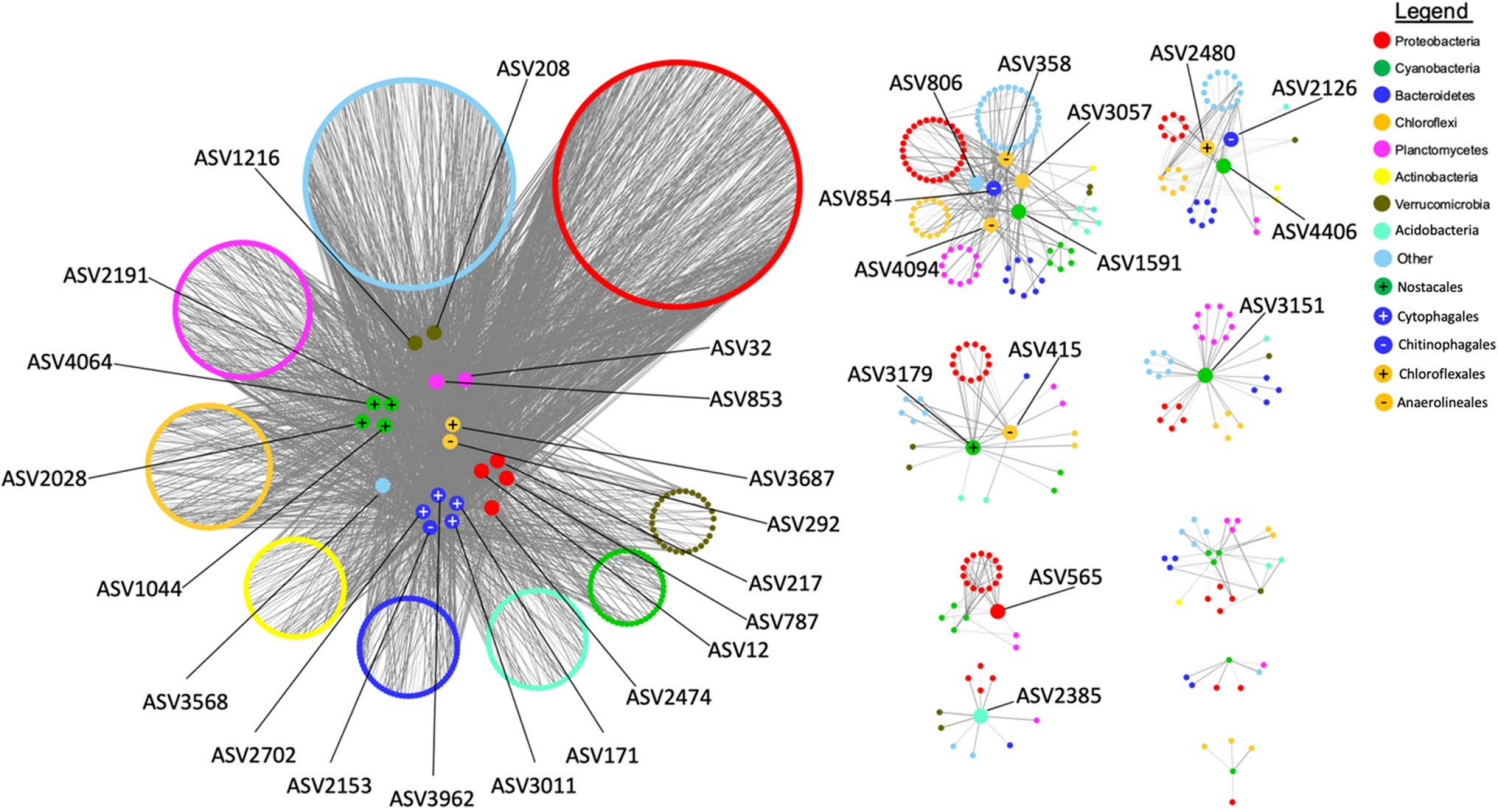
Networks based on a Spearman correlation analysis performed using short-read amplicon sequencing data. Correlation data was recruited for all ASVs with 1% or higher abundance per sample and results were then collected for any correlations with a weight of ≥ 0.85 and a *P*-value of ≤ 0.05. Results were visualized using Cytoscape by using circles to represent every ASV, line width to indicate weight of correlation, and color of each circle was used to denote phyla based on taxonomic classification previously performed. Any remaining ASV nodes that met the previously stated criteria and had 10 or more connections to other ASV nodes were centered in their respective networks as “main nodes”

While our original expectation of finding a single dominant cyanobacterial genus or species across all sites was not supported, we instead saw results similar to some fresh water hot springs of similar temperature and pH, such as those seen in Costa Rica where the most abundant cyanobacteria in each sample vary based on broader taxonomic classification such as genus [2]. We found that 11 out of the 12 samples at our site had cyanobacterial populations with an abundant filamentous cyanobacterial member. The twelfth mat had its largest abundance of cyanobacteria best classified as a unicellular cyanobacterium *Synechococcus sp*. Of the 11 most abundant filamentous cyanobacterial ASVs, all fell under the taxonomic groups of Nostocales or Leptolyngbyales. While these populations of abundant cyanobacteria are distinct, this supports the idea of cyanobacterial dominance being closely associated with their function as primary producers. The cyanobacteria presumably provide the associated communities with essential nutrients to support heterotrophic life, such as fixed carbon and possibly nitrogen [2, 42] as well as a physical substrate for attachment of other microbes. Our network analysis suggests that other abundant phyla may interact with these cyanobacteria according to their frequent co-occurrences across this study. The next most abundant top four phyla were Chloroflexi, Bacteroidetes, Proteobacteria, and Planctomycetes, as discussed below (Fig. 6)*.*

### Chloroflexi Groups Across Mat Samples

Chloroflexi from the Anaerolineae class showed high relative sequence abundance across all samples apart from sample 10A. Despite being present in all samples, these sequences have no exact match to anything isolated and taxonomically described in the NCBI database. The top hit found via an NCBI BLAST search (at 92% identity) did however match a microbe found in the microbialites of Rio Mesquites in Mexico where it was assigned a newly designated genus *Anaerolinea* and was also grown in culture at thermophilic temperatures[45] (Supplementary data Tables 1 and 2). This may be the closest known isolate to the population found at high relative proportions at Black Canyon. The source of energy for these Rio Mesquites isolates (and possibly at Black Canyon) is not thought to be phototrophy [46]. In contrast at the Yellowstone National Park alkaline hot springs, a specific genus of phototrophic Chloroflexi, *Roseiflexus*, co-exists at similar abundance levels as cyanobacteria[47].

### Diversity of Microbial Eukaryotes in the Mats

Microbial eukaryotes have been seen previously in hot springs found at a variety of pH and temperature levels [38]. In the case of the mats found in Black Canyon hot springs, a single protist ASV was abundant in 3 different samples, 9A, 9B, and 6A, and potentially has a strong influence on the prokaryotic community as suggested by the correspondence analysis. With the use of 18S rRNA gene-based high-throughput sequencing, we identified this single ASV as likely a protist from the genus *Echinamoeba* with highest identity match to *Echinamoeba thermarum*, which has regularly been seen to survive in both naturally occurring and man-made thermophilic environments[10, 38, 48].

Bacterial grazing by the amoeba *Echinamoeba* has been shown to occur when *Rhodobacter* and *Echinamoeba thermarum* were co-cultured on an agar plate[39]. The variation seen across axis 1 (Fig. 3) may further support the potential influence for selective grazing and ultimately lower diversity due to the presence of the *Echinamoeba thermarum*-like protist found only in the prokaryotic communities clustered on the left of axis 1 (samples 9A, 9B, and 6A). Those samples with any another abundant protistan member were not notably clustered in Fig. 3, based on unweighted-unifrac distance, thus implying that the novel *Echinamoeba thermarum*-like member is potentially decreasing the diversity of the prokaryotic portion of its community via grazing or other predatory actions.

Another interesting eukaryote was not a protist, but from the genus *Chaetonotus*, a poorly understood freshwater Gastrotrich that has recently been seen in other freshwater thermal systems including those that contain abundant filamentous cyanobacteria from the genus *Phormidium* [49]. In our study, this Gastrotrich was seen in samples 3A, 9C, and 13D. As seen in Fig. 3, they cluster on the opposite side of axis 1 as those with the largest abundance of the *Echinamoeba thermarum*-like protist discussed previously. Examining the abundance of this larger eukaryote in mat systems might require other sampling and analysis techniques (microscope counts) rather than those based on molecular sequences. Taken together our results provide evidence that specific grazers (e.g., *Echinamoeba* and *Chaetonotus*) may be influencing the mat’s prokaryotic microbial diversity.

We also note that an alveolate belonging to the family Eimeriidae appears to be present in multiple samples (Table 3). It was most abundant in samples 8A and 13E, found clustered closer together and separately from other samples (see Fig. 3). Based on related family members, this alveolate is possibly pathogenic in animals which may affect its abundance in the mat systems in addition to a role it may have as a grazer or bacterial consumer [50]. Our observations suggest this microbe might warrant further study to help elucidate its ecology.

### Cyanobacterial Isolates

**Fig. 7:**
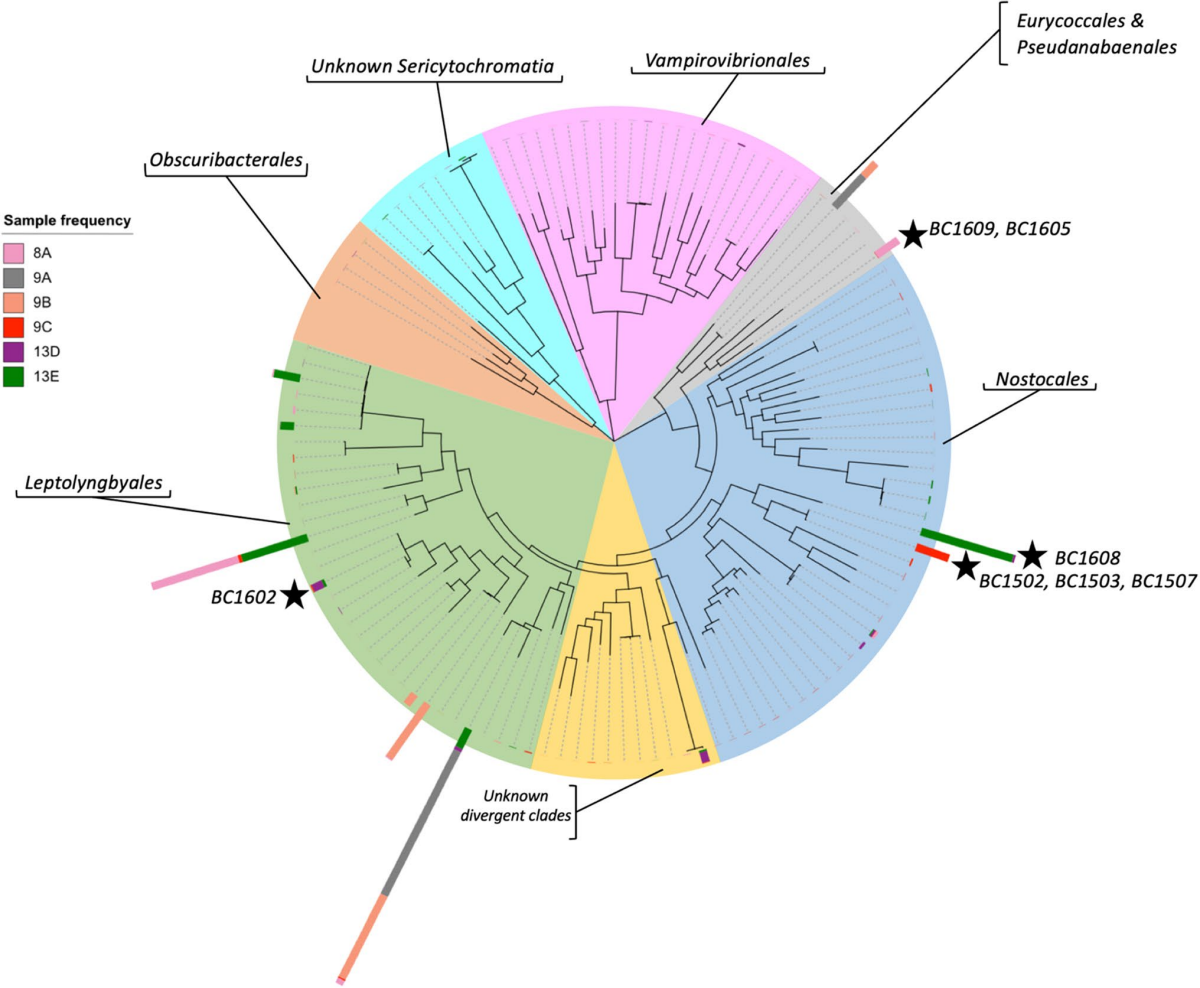
Radial phylogenetic tree based on 1400 base pair long-read sequences of 16S rRNA. The relative abundance of each cyanobacterium is represented by the height of the bar at its corresponding branch, where height equals ASV count. Stars denote isolates identified as predominant members

In the cases of mat samples 9C and 13E, we were able to culture what may be either the dominant member of these cyanobacterial communities or one very closely related to it (Fig. 7). Isolate BC1603, which was the most closely related to a *Phormidium sp*. as noted in Table 6, falls within the same monophyletic group that contains other filamentous cyanobacteria such as the strain *Microcoleus PCC 8701* seen in stromatolite structures in thermophilic freshwater environments in Yellowstone National Park, USA, at 56 °C and *Phormidium sp. NgrPH08*, most recently recorded living in the hot springs of Greece between 26 and 58 °C and also near circumneutral pH (Supplementary Fig. 2) [51, 52]. Despite being extremely distant environments geographically, our isolate BC1603 also has close relatives at the 16S rRNA level to dominant members of microbial mats found in Spain and Thailand where in some cases the top layers of these mats were characterized as being a monoculture of *Phormidium* and *Microcoleus* using microscopy techniques [53, 54]. We also isolated another *Phormidium* BC1608 that is demonstrably abundant in our system.

Isolates BC1502, BC1503, BC1507, and BC1604 showed a 97% nucleotide identity to the strain Lao26 from the newly established genus *Aerosakkonema funiforme*[40]. Although not previously found in a thermal-microbial mat environment, these filamentous cyanobacteria were found in a fresh water reservoir and were reportedly grown in the laboratory at temperatures up to 35 °C, nearing the thermophilic range[40]. Additionally, *Aerosakkonema sp.* were characterized as having a gas vacuole which in a microbial mat setting may help them to rise to the top of the mat where light conditions may be better suited for optimal growth. Other taxa closely related to *Aerosakkonema funiforme* were until now thought to mainly be distributed within Asia.

### Correlation Analysis of Abundant Prokaryotes

Our network analysis showed that our dominant cyanobacteria in each mat were often associated with Chloroflexi and Bacteroidetes. For example, the network for samples 13A–B (Fig. 6A) has cyanobacterial nodes from the order Nostocales and these cyanobacteria may be nitrogen fixing based on similar investigations performed in the past [2, 43]. Their potential ability to fix nitrogen could fulfill the nitrogen demand of this environment given the low abundance of *Fischerella* and *Mastigocladus* sp. cyanobacteria mat species that typically fix nitrogen [55].

Four of the five main Bacteroidetes nodes in this network belonged to the order Cytophagales and the highest classification assigned being ASV3011 as OLB12 sp. from the group *Microscillaceae*, recently described as being chemoorganotrophs and strict aerobes, thus requiring oxygen, either from photosynthetic members or from the atmosphere, and using the substrates being exuded by other community members [56]. Some members of the Bacteroidetes group may also be involved in nitrogen cycling [57]. In addition the main Bacteroidetes node found in the network seen in Fig. 6B was classified as belonging to the family *Saprospiraceae*, best known for also containing several strains belonging to the genus *Saprospira*, previously seen lysing cyanobacteria while also lacking the ability to synthesize nine different essential amino acids [58, 59]. While these predatory species are seen in marine-based systems, the lack of “terrestrial” *Saprospira* studies may indicate that this group may be overlooked and is occurring in hot spring habitats potentially as microbes that could be influencing the microbial diversity of some mat communities.

Of the two Chloroflexi main nodes, the most abundant belonged to the sub-group SBR1031 which has been described as an anaerobic microbe capable of catalyzing cellulose hydrolysis and using its type VI pili for adhesion during cellular aggregation which in this case could prevent cell loss due to stream flow [60]. A second set of Chloroflexi main node ASVs was classified as belonging to the order Chloroflexales, a better studied anoxygenic and phototrophic bacterium in hot springs[61]. Co-occurrence of Cyanobacteria and Chloroflexi has been previously described such as in hot spring environments at Yellowstone National Park[47]. Their co-occurrence supports the idea of the Black Canyon mat community possibly being energetically reliant on both these two major groups of bacteria based on meta-transcriptomic studies at other sites [42, 62]. Metagenomic genome assemblies and meta-transcriptomic studies at the Black Canyon would be useful to untangle the metabolic interconnections of Cyanobacteria, Chloroflexi, and Bacteroidetes implied by our network analysis.

## Supplementary Information

ESM 1: Supplementary Figure 1. Rarefaction curves representing the alpha diversity of the prokaryotic community from each sample by measure of ASVs observed. The alpha diversity seen at a subsampling of 6000 sequences for all samples, both short amplicon (solid line) and full-length (dashed line) marker gene, shows varying number of ASVs observed at equal depths. (DOCX 415 kb)

ESM 2: Supplementary Figure 2. Neighbor-joining phylogenetic tree of isolates. Tree is based on Jukes-Cantor distances that includes all cyanobacteria isolated from Black Canyon mats. Bold branches indicate bootstrap values at 50% or higher after 1,000 replicates. (DOCX 752 kb)

ESM 3: Supplementary Table 1 (TXT 1886 kb)

ESM 4: Supplementary Table 2 (TXT 4223 kb)

ESM 5: Supplementary Table 3 (TXT 81 kb)

## Data Availability

Raw sequencing data is available on NCBI within BioProject accession number PRJNA883542. Partial 16S rRNA sequences for isolated cyanobacteria can be found under accession numbers OP739457-OP739474. The datasets generated during and/or analyzed during the current study are available in Supplementary tables and in the NCBI repositories mentioned above.

## References

[1] Rothschild LJ, Mancinelli RL (2001). Life in extreme environments. Nature.

[2] Uribe-Lorío L, Brenes-Guillén L, Hernández-Ascencio W et al (2019) The influence of temperature and pH on bacterial community composition of microbial mats in hot springs from Costa Rica. Microbiologyopen 1–26. 10.1002/mbo3.893 10.1002/mbo3.893 PMC6813449 31271524

[3] Tang J, Liang Y, Jiang D (2018). Temperature-controlled thermophilic bacterial communities in hot springs of western Sichuan, China. BMC Microbiol.

[4] Wang S, Hou W, Dong H et al (2013) Control of temperature on microbial community structure in hot springs of the Tibetan Plateau. PLoS One 8. 10.1371/journal.pone.0062901 10.1371/journal.pone.0062901 PMC3647046 23667538

[5] Papke RT, Ramsing NB, Bateson MM, Ward DM (2003). Geographical isolation in hot spring cyanobacteria. Environ Microbiol.

[6] Inskeep WP, Jay ZJ, Tringe SG (2013). The YNP metagenome project: Environmental parameters responsible for microbial distribution in the Yellowstone geothermal ecosystem. Front Microbiol.

[7] Bartram AK, Lynch MDJ, Stearns JC (2011). Generation of multimillion-sequence 16S rRNA gene libraries from complex microbial communities by assembling paired-end Illumina reads. Appl Environ Microbiol.

[8] Pace NR, Stahl DA, Lane DJ, Olsen GJ, Marshall KC (1986). The analysis of natural microbial populations by ribosomal RNA sequences. Advances in microbial ecology. Advances in microbial ecology.

[9] Lane DJ, Pace B, Olsen GJ (1985). Rapid determination of 16S ribosomal RNA sequences for phylogenetic analyses. Proc Natl Acad Sci U S A.

[10] Brown PB, Wolfe GV (2006). Protist genetic diversity in the acidic hydrothermal environments of Lassen Volcanic National Park, USA. J Eukaryot Microbiol.

[11] Bowers RM, Nayfach S, Schulz F (2022). Dissecting the dominant hot spring microbial populations based on community-wide sampling at single-cell genomic resolution. ISME J.

[12] Reichart NJ, Bowers RM, Woyke T, Hatzenpichler R (2022). Metagenomes and metagenome-assembled genomes from substrate-amended hot spring sediment incubations from Yellowstone National Park. Microbiol Resour Announc.

[13] Jarett JK, Džunková M, Schulz F (2020). Insights into the dynamics between viruses and their hosts in a hot spring microbial mat. ISME J.

[14] Elke Jaspers and Jorg Overmann (2004). Ecological significance of microdiversity: identical 16S rRNA gene sequences can be found in bacteria with highly divergent genomes and ecophysiologies. Appl Environ Microbiol.

[15] Callahan BJ, McMurdie PJ, Rosen MJ (2016). DADA2: High-resolution sample inference from Illumina amplicon data. Nat Methods.

[16] Hug LA, Baker BJ, Anantharaman K (2016). A new view of the tree of life. Nat Microbiol.

[17] Moran MJ, Wilson JW, Beard LS (2015). Hydrogeology and sources of water to select springs in Black Canyon, south of Hoover Dam, Lake Mead National Recreation Area. Scientific investigations report 2015-5130.

[18] (2020) Black Canyon Springs. https://www.nps.gov/lake/learn/nature/black-canyon-springs.htm.

[19] (2012) Naegleria fowleri, A Public Health Risk. http://npshistory.com/publications/lake/naegleria.pdf.

[20] Thiel V, Wood JM, Olsen MT (2016). The dark side of the mushroom spring microbial mat: Life in the shadow of chlorophototrophs. I. Microbial diversity based on 16S rRNA gene amplicons and metagenomic sequencing. Front Microbiol.

[21] Ward DM, Weller R, Bateson MM (1990). 16S rRNA sequences reveal numerous uncultured inhabitants in a well-studied natural community. Nat.

[22] Castenholz RW (1969). Thermophilic blue-green algae and the thermal environment. Bacteriol Rev.

[23] Thompson LR, Sanders JG, McDonald D, et al (2017) A communal catalogue reveals Earth’s multiscale microbial diversity. Nature 551:457–463. https://doi.org/10.1038/nature24621 10.1038/nature24621 PMC6192678 29088705

[24] Walters W, Hyde ER, Berg-lyons D (2015). Transcribed spacer marker gene primers for microbial community surveys. mSystems.

[25] Amaral-Zettler LA, McCliment EA, Ducklow HW, Huse SM (2009). A method for studying protistan diversity using massively parallel sequencing of V9 hypervariable regions of small-subunit ribosomal RNA Genes. PLoS One.

[26] Stoeck T, Bass D, Nebel M (2010). Multiple marker parallel tag environmental DNA sequencing reveals a highly complex eukaryotic community in marine anoxic water. Mol Ecol.

[27] Callahan BJ, Grinevich D, Thakur S (2021). Ultra-accurate microbial amplicon sequencing directly from complex samples with synthetic long reads. Microbiome.

[28] Bolyen E, Rideout JR, Dillon MR (2019). Reproducible, interactive, scalable and extensible microbiome data science using QIIME 2. Nat Biotechnol.

[29] Frank JA, Reich CI, Sharma S (2008). Critical evaluation of two primers commonly used for amplification of bacterial 16S rRNA genes. Appl Environ Microbiol.

[30] Glöckner FO, Yilmaz P, Quast C (2017). 25 years of serving the community with ribosomal RNA gene reference databases and tools. J Biotechnol.

[31] Guillou L, Bachar D, Audic S (2013). The Protist Ribosomal Reference database (PR2): a catalog of unicellular eukaryote Small Sub-Unit rRNA sequences with curated taxonomy. Nucleic Acids Res.

[32] Lozupone C, Knight R (2005). UniFrac: a new phylogenetic method for comparing microbial communities. Appl Environ Microbiol.

[33] Vázquez-Baeza Y, Pirrung M, Gonzalez A, Knight R (2013). EMPeror: a tool for visualizing high-throughput microbial community data. Gigascience.

[34] Faust K, Raes J (2016). CoNet app: Inference of biological association networks using Cytoscape [version 1; referees: 2 approved with reservations]. F1000Res..

[35] Mary Mennes Allen (1968). Simple conditions for growth of unicellular blue-green algae on plates. J of Phyc..

[36] Nübel U, Garcia-Pichel F, Muyzer G (1997). PCR primers to amplify 16S rRNA genes from cyanobacteria. Appl Environ Microbiol.

[37] Altschul SF, Gish W, Miller W (1990). Basic local alignment search tool. J Mol Biol.

[38] Oliverio AM, Power JF, Washburne A (2018). The ecology and diversity of microbial eukaryotes in geothermal springs. ISME J.

[39] Baumgartner M, Yapi A, Gröbner-Ferreira R, Stetter KO (2003). Cultivation and properties of *Echinamoeba thermarum* n. sp., an extremely thermophilic amoeba thriving in hot springs. Extremophiles.

[40] Thu NK, Tanabe Y, Yoshida M (2012). Aerosakkonema funiforme gen. et sp. nov. (Oscillatoriales), a new gas-vacuolated oscillatorioid cyanobacterium isolated from a mesotrophic reservoir. Phycologia.

[41] Miller SR, Castenholz RW, Pedersen D (2007). Phylogeography of the thermophilic cyanobacterium *Mastigocladus laminosus*. Appl Environ Microbiol.

[42] Klatt CG, Wood JM, Rusch DB (2011). Community ecology of hot spring cyanobacterial mats: predominant populations and their functional potential. ISME J.

[43] Finsinger K, Scholz I, Serrano A (2008). Characterization of true-branching cyanobacteria from geothermal sites and hot springs of Costa Rica. Environ Microbiol.

[44] Alcorta J, Espinoza S, Viver T (2018). Temperature modulates *Fischerella thermalis* ecotypes in Porcelana Hot Spring. Syst Appl Microbiol.

[45] Corman JR, Poret-Peterson AT, Uchitel A, Elser JJ (2016). Interaction between lithification and resource availability in the microbialites of Río Mesquites, Cuatro Ciénegas, México. Geobiology.

[46] Sekiguchi Y, Yamada T, Hanada S (2003). Anaerolinea thermophila gen. nov., sp. nov. and *Caldilinea aerophila* gen. nov., sp. nov., novel filamentous thermophiles that represent a previously uncultured lineage of the domain bacteria at the subphylum level. Int J Syst Evol Microbiol.

[47] Bennett AC, Murugapiran SK, Hamilton TL (2020). Temperature impacts community structure and function of phototrophic Chloroflexi and Cyanobacteria in two alkaline hot springs in Yellowstone National Park. Environ Microbiol Rep.

[48] Valster RM, Wullings BA, van den Berg R, van der Kooij D (2011). Relationships between free-living protozoa, cultivable *Legionella* spp., and water quality characteristics in three drinking water supplies in the Caribbean. Appl Environ Microbiol.

[49] Della Porta G, Hoppert M, Hallmann C (2022). The influence of microbial mats on travertine precipitation in active hydrothermal systems (Central Italy). Depos Rec.

[50] Yang R, Brice B, Ryan U (2016). Morphological and molecular characterization of *Eimeria purpureicephali* n. sp. (Apicomplexa: Eimeriidae) in a red-capped parrot (Purpureicephalus spurius, Kuhl, 1820) in Western Australia. Int J Parasitol Parasites Wildl.

[51] Bravakos P, Kotoulas G, Skaraki K (2016). A polyphasic taxonomic approach in isolated strains of Cyanobacteria from thermal springs of Greece. Mol Phylogenet Evol.

[52] Pepe-Ranney C, Berelson WM, Corsetti FA (2012). Cyanobacterial construction of hot spring siliceous stromatolites in Yellowstone National Park. Environ Microbiol.

[53] Grimalt JO, de Wit R, Teixidor P, Albaigés J (1992). Lipid biogeochemistry of *Phormidium* and *Microcoleus* mats. Org Geochem.

[54] Thummajitsakul S (2012). Antibacterial activity of crude extracts of cyanobacteria *Phormidium* and *Microcoleus* species. African J Microbiol Res.

[55] Miller SR, Purugganan MD, Curtis SE (2006). Molecular population genetics and phenotypic diversification of two populations of the thermophilic cyanobacterium *Mastigocladus laminosus*. Appl Environ Microbiol.

[56] Hahnke RL, Meier-Kolthoff JP, García-López M et al (2016) Genome-based taxonomic classification of Bacteroidetes. Front Microbiol 7. 10.3389/fmicb.2016.02003 10.3389/fmicb.2016.02003 PMC5167729 28066339

[57] Herrmann M, Rusznyák A, Akob DM (2015). Large fractions of CO2-fixing microorganisms in pristine limestone aquifers appear to be involved in the oxidation of reduced sulfur and nitrogen compounds. Appl Environ Microbiol.

[58] Shi M, Zou L, Liu X, Gao Y, Zhang Z, Weizhong W, Wen D, Zhangliang Chen CA (2006). A novel bacterium *Saprospira* sp. strain PdY3 forms bundles and lyses cyanobacteria. Front Biosci.

[59] Saw JHW, Yuryev A, Kanbe M (2012). Complete genome sequencing and analysis of *Saprospira grandi*s str. Lewin, a predatory marine bacterium. Stand Genomic Sci.

[60] Xia Y, Wang Y, Wang Y (2016). Cellular adhesiveness and cellulolytic capacity in Anaerolineae revealed by omics-based genome interpretation. Biotechnol Biofuels.

[61] Grouzdev DS, Kuznetsov BB, Keppen OI (2015). Reconstruction of bacteriochlorophyll biosynthesis pathways in the filamentous anoxygenic phototrophic bacterium *Oscillochloris trichoides* DG-6 and evolution of anoxygenic phototrophs of the order Chloroflexales. Microbiol (United Kingdom).

[62] Liu Z, Klatt CG, Wood JM (2011). Metatranscriptomic analyses of chlorophototrophs of a hot-spring microbial mat. ISME J.

