# Supplementary figures and images for: Diverse Microbial Hot Spring Mat Communities at Black Canyon of the Colorado River
Source: Microb Ecol. 2023 Feb 9;86(3):1534–51. doi: 10.1007/s00248-023-02186-x (PMC10497668; doi:10.1007/s00248-023-02186-x)

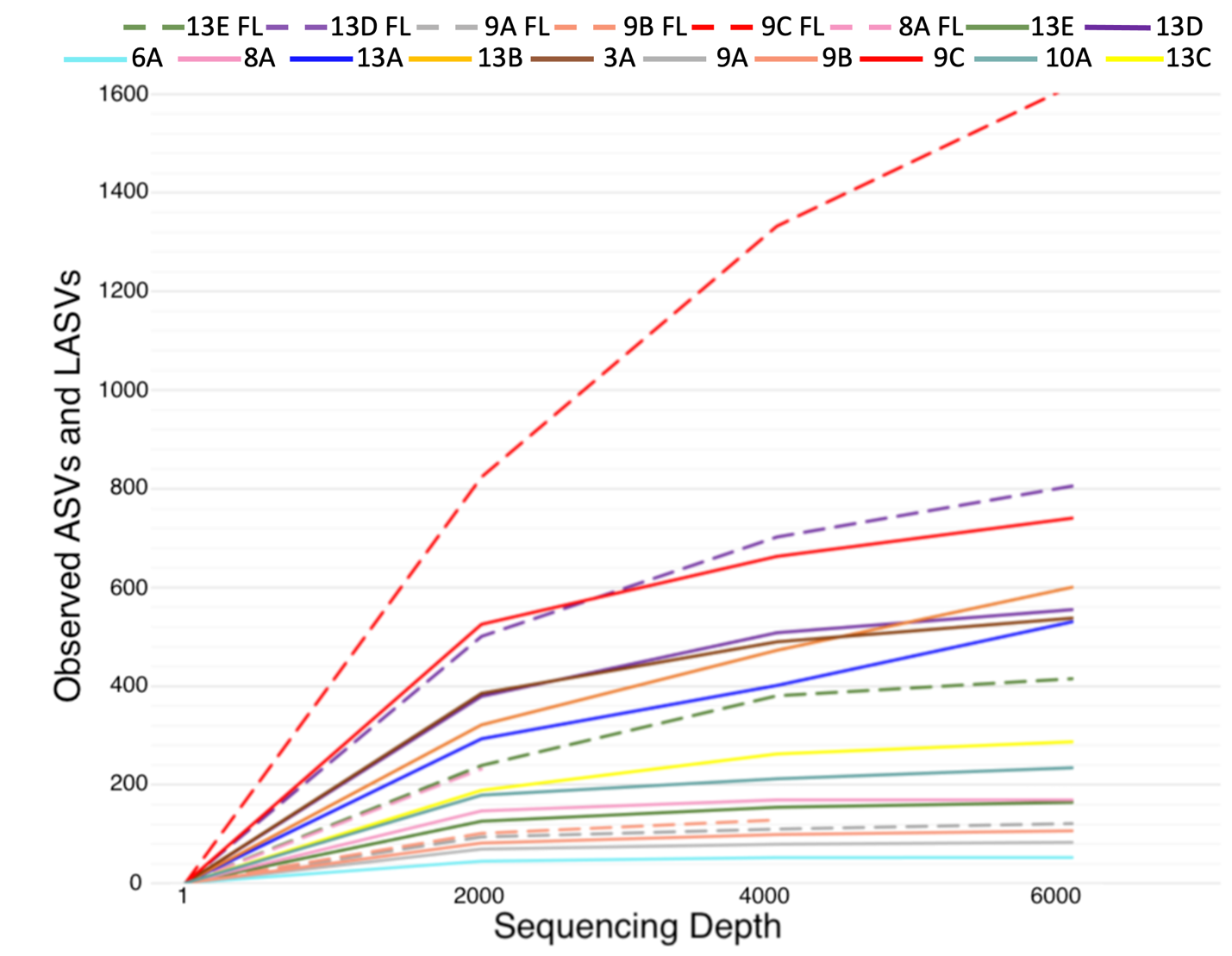

Supplement: Supplementary file 1 — Supplementary Figure 1. Rarefaction curves representing the alpha diversity of the prokaryotic community from each sample by measure of ASVs observed. The alpha diversity seen at a subsampling of 6000 sequences for all samples, both short amplicon (solid line) and full-length (dashed line) marker gene, shows varying number of ASVs observed at equal depths. (DOCX 415 kb) [file 248_2023_2186_MOESM1_ESM.docx]

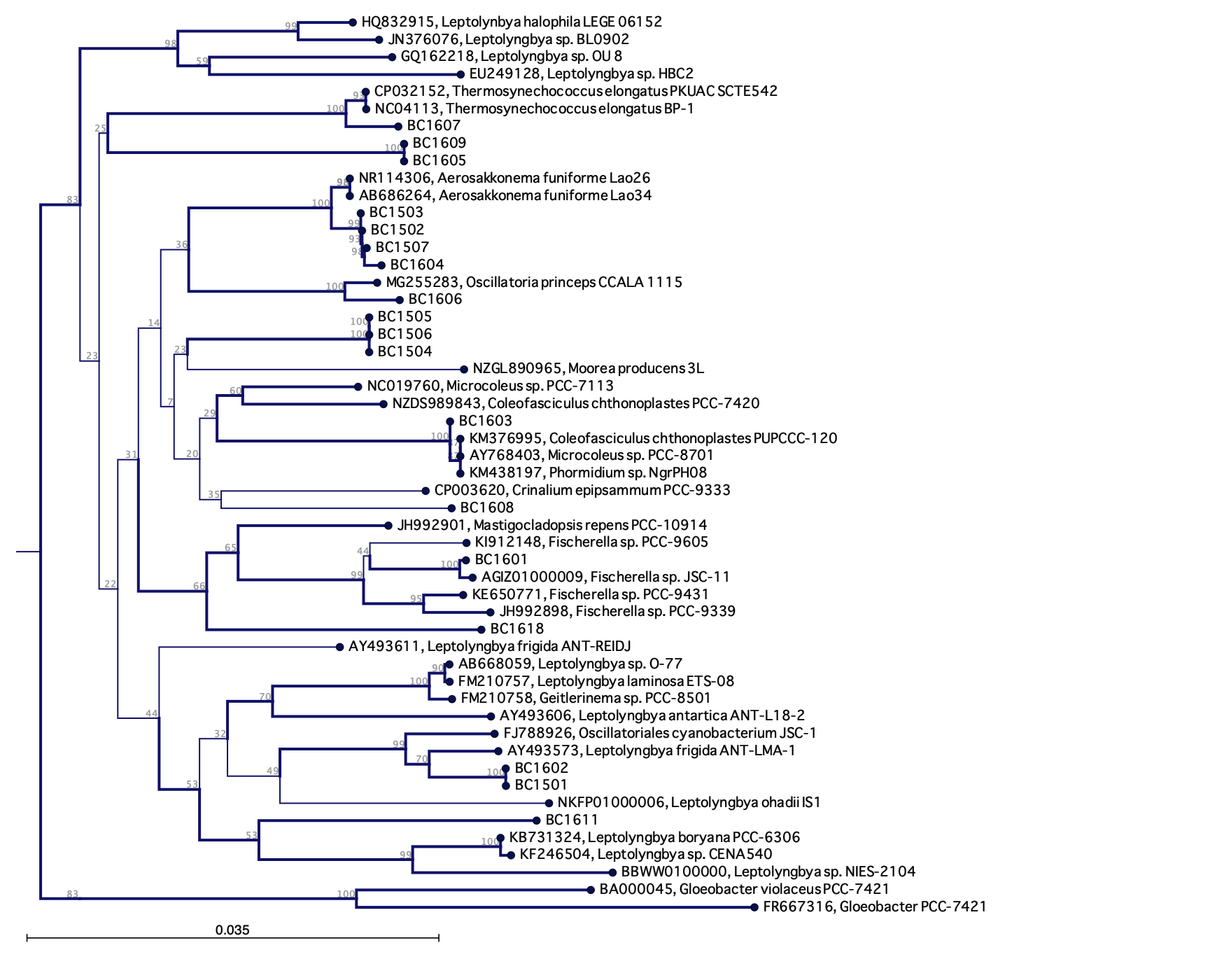


Legend

Site 3

Site 6

Site 8

Site 9

Site 10

Site 13

Supplement: Supplementary file 2 — Supplementary Figure 2. Neighbor-joining phylogenetic tree of isolates. Tree is based on Jukes-Cantor distances that includes all cyanobacteria isolated from Black Canyon mats. Bold branches indicate bootstrap values at 50% or higher after 1,000 replicates. (DOCX 752 kb) [file 248_2023_2186_MOESM2_ESM.docx]
